# Clustering-enhanced stock price prediction using deep learning

**DOI:** 10.1007/s11280-021-01003-0

**Published:** 2022-04-14

**Authors:** Man Li, Ye Zhu, Yuxin Shen, Maia Angelova

**Affiliations:** 1 School of IT, Deakin University, Geelong, Australia; 2 College of Intelligence and Computing, Tianjin University, Tianjin, China

**Keywords:** Financial data analytics, Stock prediction, Clustering-enhanced deep learning

## Abstract

In recent years, artificial intelligence technologies have been successfully applied in time series prediction and analytic tasks. At the same time, a lot of attention has been paid to financial time series prediction, which targets the development of novel deep learning models or optimize the forecasting results. To optimize the accuracy of stock price prediction, in this paper, we propose a clustering-enhanced deep learning framework to predict stock prices with three matured deep learning forecasting models, such as Long Short-Term Memory (LSTM), Recurrent Neural Network (RNN) and Gated Recurrent Unit (GRU). The proposed framework considers the clustering as the forecasting pre-processing, which can improve the quality of the training models. To achieve the effective clustering, we propose a new similarity measure, called Logistic Weighted Dynamic Time Warping (LWDTW), by extending a Weighted Dynamic Time Warping (WDTW) method to capture the relative importance of return observations when calculating distance matrices. Especially, based on the empirical distributions of stock returns, the cost weight function of WDTW is modified with logistic probability density distribution function. In addition, we further implement the clustering-based forecasting framework with the above three deep learning models. Finally, extensive experiments on daily US stock price data sets show that our framework has achieved excellent forecasting performance with overall best results for the combination of Logistic WDTW clustering and LSTM model using 5 different evaluation metrics.

## Introduction

With the explosive growth and development of big data technologies, the power of exploring real-world phenomenon has been greatly enhanced and evolved over past two decades. Undoubtedly, the economic and financial time series forecasting has become one of the most attractive topics for many parties of our society. Especially, the outbreak of unexpected events, such as worldwide COVID-19, makes financial time series forecasting more difficult and challenging. Thus, existing time series forecasting methods, decision making strategies and even understanding about the predictability need further investigation [33, 43]. A growing number of financial forecasting studies have employed deep learning models to forecast stock prices in recent years [38]. In particular, compared to other types of neural networks, such as Convolutional Neural Networks (CNNs) and Deep Belief Networks (DBNs), LSTM networks have achieved superior performance in stock forecasting due to its ideal fitness of nonlinear modeling [16, 20]. Recurrent Neural Networks (RNNs) and Gated Recurrent Units (GRUs) are the other two commonly used deep learning models for stock return prediction [11, 41].

However, we find that most existing stock price prediction studies have chosen either some widely used market index time series or time series data for only a few stocks. For example, Lee and Yoo [27] use monthly price data for 10 stocks in S&P 500 to predict prices with LSTM, RNN and GRU models. Hiransha et al. [19] use 3 Indian stocks and 2 US stocks to train deep learning models and predict stock prices. The small sample size and limited number of stocks used in previous studies motivate us to examine how deep learning models perform to predict stock prices with sufficient price time series data. Nevertheless, some studies have used a large price time series dataset to test the predictive ability of LSTM model. For example, Fischer, and Krauss [15] apply LSTM model to predict stock returns with S&P 500 components price data. This most comparable study further motivates us to examine whether their forecasting results are reliable as they use random forest, deep net, and logistic regression as benchmark models to compare the performance of LSTM. If other deep learning models, such as RNN and GRU, are used as comparable models in prediction framework, the forecasting results can be more convincing.

More importantly, we also find that in some time series forecasting studies, clustering has been incorporated into prediction framework, which has led to a better forecasting performance. For instance, Zhang et al.[49] use *k*-means clustering to forecast passenger flow. Zhou et al. [50] use density-based spatial clustering to eliminate abnormal noises and data groups, and implement LSTM model to predict wind power spot. These relevant time series forecasting studies in other scientific domains further motivate us to apply a clustering-enhanced prediction framework to financial time series data. Therefore, we propose a clustering-enhanced prediction framework enhanced with new clustering similarity measure and deep learning models in this study.

In order to implement an effective clustering method, we propose a new similarity measure called Logistic Weighted Dynamic Time Warping (LWDTW) to calculate distance between stock price time series. For comparison purpose, we use two well-known distance functions, Euclidean distance and standard Dynamic Time Warping (DTW) method, as two benchmark similarity measures [14, 37]. The key insight of LWDTW is to design a new weight function to give favour or penalty to different stock return observations when calculating distance matrix. By using individual US stock price data, we find that the distribution of stock returns is not a normal distribution as there are much more extreme return observations than they should be in a normally distributed data. This is consistent with empirical findings of stock price time series characteristics, such as dynamic, non-stationary, nonlinear and chaos. Furthermore, their empirical distributions are better described by logistic distribution probability density function with higher peaks and fatter tails. Thus, the new proposed Logistic WDTW measure incorporates logistic distribution probability density function as the weight function to underweight those extremely high or low return observations but give more weights to normal return observations when calculating distance matrices. That is, the more similar the return patterns are, the more similar stocks are. After that, we integrate the clustering into the proposed framework for training the LSTM, RNN and GRU models and predicting the stock prices. To show the effect of clustering, we also implement the three prediction models without the consideration of clustering, i.e., we apply LSTM, RNN and GRU models to the stock price time series directly.

The contributions of this study are summarised as follows:

- Based on the empirical distribution of stock return time series, we propose a novel LWDTW similarity measure with logistic distribution probability density function, which can cluster the similar stocks effectively.
- We develop a novel clustering-enhanced deep learning framework for predicting the stock prices. It integrates LWDTW clustering with three widely-used deep learning models, LSTM, RNN, and GRU.
- We conduct extensive experimental evaluation on real stock data and demonstrate the performance of different prediction models within the framework using five well-known evaluation metrics.

The remainder of this paper is organised as follows. Section 2 discusses the most relevant works, including distance based similarity, clustering algorithms, deep learning forecasting techniques, and their applications in financial time series mining. Section 3 presents the problem statement and the proposed framework with the detailed modules. Section 4 describes the data selection for this study and the data pre-processing information. Finally, we provide the experimental setting and results in Section 5, and conclude this study in Section 6, respectively.

## Related work

In this section, many related works to this study are reviewed, such as research about distance-based similarity measures, papers involved clustering into forecasting time series and those well-known deep learning forecasting models.

### Distance-based similarity measures

When conducting clustering based strategy to forecast, the first step is to calculate similarities between stock price time series and then assign individual stocks into clusters based on their similarities. This step of clustering could be used as data pre-processing for the forecasting process. The right choice of similarity measures is the critical and fundamental work for the effective implementation of clustering algorithms. Generally, there are four types of similarity measures in the literature, including distance-based, compression-based, feature-based and model-based measures in [25, 39, 42].

Distance-based functions are suitable to capture similarities between short time series [1], which can be divided into two categories, non-elastic *L*_*p*_ norm distance and elastic distance functions. The first category mainly include Manhattan distance (p = 1), Euclidean distance (p = 2) and Chebyshev distance (p=$\infty $), and Euclidean distance is recognized as the most useful and popular measure among them [14]. It is obviously seen that those non-elastic functions are easily implemented with low computational cost, but they are not able to capture similarities between unequal length time series due to the ”non-elastic” characteristics of time axis. In the second category, distance functions have overcome the shortcomings of the ”non-elastic” measures by allowing to warp time axis and finding the smallest distance between unequal length or mismatching time series. In [6] and [37], authors have addressed that Dynamic time warping (DTW) is the most well-known elastic measure and good at dealing with numerical time series . EDIT Distance, Edit Distance with Real Penalty (ERP), Edit Distance on Real Sequence (EDR) and the Longest Common Subsequence (LCSS) measures are also proposed and used to capture similarities based on similarity scores with a threshold *𝜖* [2, 5, 34].

Compared to non-elastic measures, elastic measures, such as DTW, are more complicated and computationally time consuming. Thus, on the one hand, in order to improve computational efficiency, two significant methods Sakoe–Chiba [35] band and the Itakura Parallelogra [21] have been proposed that can restrict sliding window lengths and reduce the number of comparisons. On the other hand, the standard DTW has been developed to apply in real-world data mining tasks. By relaxing the ideal assumption of standard DTW, one advanced DTW model, called weighted DTW (WDTW), has been proposed to recognize the relative importance of different alignments when exploring optimal warping path, where different weight functions are considered in different scenarios. For instance, Jeong et al. [22] first proposed the idea of WDTW and use the modified logistic cumulative distribution function to assign different weights for neighbouring points and farther points. Oregi et al. [31] also propose a forgetting mechanism based on a memory function as weight function for WDTW to recognize the relative importance of recent observations and ignore the contributions of earlier observations in on-line learning scenarios.

In this study, we further extend the existing WDTW literature to financial time series domain by modifying weight functions with logistic distribution probability density function. The novel proposed logistic WDTW (LWDTW) similarity measure, as well as benchmark Euclidean distance and standard DTW, is used to calculate distance matrices between individual stock price time series.

Regarding clustering methods, partitioning methods are the most widely used and popular, depending on how to define the centre point of each group. *K*-means and *k*-medoids methods are included in this category [24, 29, 30]. Hierarchical clustering is useful to group observations at different levels, especially for data visualization [23]. Density-based and grid-based methods also perform well for other data types, such as non-spherical, arbitrary data, or grid-structure data [13, 44]. Due to the characteristics of financial time series data, *k*-medoids clustering method is well suited to group stocks based on their distance matrices with little influences of extreme prices. Therefore, we select *k*-medoids clustering method to identify similar stocks with similar price patterns in this study.

### Deep learning prediction framework with clustering involvement

Deep learning prediction framework with clustering involvement has been successfully applied in many real-world time series analysis. For example, Zhang et al. [49] use two-step *k*-means clustering model before the implementation of LSTM model to forest passenger flow. They use Pearson’s correlation coefficient to capture variation trends about passenger flow of main classes and ridership volume information of each subclass within one main class and then form sub-classes for further passenger flow predictions with LSTM model. Dong et al. [9] utilize *k*-means method to subdivide the obtained datasets into training sets and test sets, and then implement the CNN model to forecast short-term electricity load. In another study, Zhou et al. [50] initially adopt density-based spatial clustering to eliminate abnormal noises and data groups, then form different clusters of wind impact factors based on *k*-means clustering, finally use LSTM to predict wind power for each cluster. Therefore, clustering has been incorporated into deep learning prediction framework with a significant achievement in many scientific domains, but little previous research has been done for financial time series forecasting. The study is going to fill this gap by doing stock price forecasting with clustering-enhanced prediction framework in different settings.

In addition, regarding the deep learning applications in financial time series, there are two recent survey papers to mention. Ozbayoglu et al. [32] have presented a number of studies that implement deep learning models in different financial applications, such as asset price forecasting, risk assessment, fraud detection, and portfolio management. The survey paper of Sezer et al. [38] is focused on the topic of forecasting application with deep learning techniques for financial time series where a number of studies in financial applications with deep learning models are presented. For instance, price data of 3 Indian stocks and 2 US stocks are used to train deep learning models and predict stock prices in [19]. Using 10 stocks in the S&P 500, Lee et al. [27] forecast monthly returns with RNN, LSTM and GRU models. Their main purpose is to construct threshold portfolios to realise risk-return target by using forecasting results to help with portfolio selection. Deszi and Nnistor [8] use Romanian stock market index data to compare the forecasting performances of LSTM and RNN models. Samarawickrama et al. [36] compare predictive abilities among Simple RNN, RNN, LSTM as well as GRU models with stock price data from Colombo Stock Exchange. Using S&P 500 components price data, Krauss et al. [26] have examined profitability of portfolios that are constructed on the forecasting results of deep neural networks, gradient-boosted-trees, random forests and ensembles.

This further motivates us to investigate whether deep learning models can perform better in predicting stock prices with sufficient price data and whether clustering-enhanced prediction framework can provide more accurate forecasting results, compared to no clustering setting in the most relevant existing studies.

### Deep learning forecasting models for time series

In terms of time series or sequential data, RNN and LSTM are the most widely used DL methods [3]. As the first algorithm that can remember its inputs by using internal memory, RNN is different from the well-known Feedforward Neural Network (FFNN) [48]. In FFNN, information flows in one direction where input data is processed in hidden layers, then output value is produced. For instance, in CNN, a commonly used type of FFNN, an image content data is processed in input and hidden units, then its classification results will be produced at the output end. Nevertheless, as units in RNN hidden layers have internal memory by storing historic information, each RNN unit takes not only current input data but previous data at the same time to make output values. That is, output values in RNN are dependent on both historic and current input sequences, which enables RNN to be suitable for prediction mining tasks. Therefore, compared with other DL algorithms, RNN can provide users with a much deeper understanding of sequential data like time series data, such as videos, audios, texts, financial data, weather data, etc.

Long-short term memory (LSTM) is a special type of RNN model. The main difference between RNN and LSTM is that LSTM can remember both short and long term relevant information and avoid the long-term dependency problem of conventional RNN [16, 20]. In other words, when the gap between the relevant historic information and current point of time is very large, RNN is not able to connect this information to current task because of its vanishing gradient and exploding gradient problem [46]. While, LSTM is designed to solve this problem with LSTM units consisting of cells with input gates, forget gates and output gates. Relevant and useful information is selected by these gates, stored for a long time period and connected to current task when it is required [3]. Many previous studies show that LSTM model has achieved incredible success in many time series application, compared to conventional RNN, including natural language processing, speech recognition, financial forecasting, etc [17, 18]. Although there are other types of deep learning models, like graph convolutional networks [40, 47] and representation learning techniques [4, 28], they are designed for processing the data with complex structures, systems and even interaction information from different sources.

Therefore, in this study, we use LSTM model to forecast stock prices. For comparison purpose, both RNN model and GRU model are also selected to do forecasting.

## Problem and methodology

### Problem statement

Traditional finance prediction framework has been built on some ideal assumptions of financial theory, such as investors’ risk-averse preferences, homogeneous investment beliefs, no transaction costs or unlimited access to risk-free assets, etc. Portfolio sorts and linear regressions are used as the main forecasting approaches. Thus, applying deep learning techniques in financial forecasting is an emerging research trend. The main advantage of such forecasting is that it doesn’t rely on the above strict assumptions and doesn’t require too much assistance from financial experts. Deep learning techniques are able to extract high-level useful features and patterns of input data automatically. Therefore, this paper is going to fill this gap by employing efficient clustering-enhanced framework with 4 different settings to achieve excellent prediction performances of stock prices.

#### **Definition 1**

Stock price time series data is represented as a sequence of prices over *t* time intervals, denoted as *P* = {*p*_1_,*p*_2_,...,*p*_*t*_}, where *p*_1_ represents stock price at the end of first time interval, and *p*_*t*_ represents stock price for the last time interval, *t* is the length of *P*.

In this study, we obtain raw daily price time series data and then transform raw prices into normalised prices by using the following method. First, we take min-max transformation to get the time series into [*min*,*max*] range, $\hat {P}=\{\hat {p_{1}}, \hat {p_{2}}, ..., \hat {p_{t}}\}$ with (1):

1 $$ \hat{p_{t}}=2\frac{p_{t}-p_{t}.\min}{p_{t}.\max-p_{t}.\min}-1,  $$

where $p_{t}.\min \limits $ and $p_{t}.\max \limits $ are the minimum and maximum prices of the stock price time series *P* respectively. When processing price prediction task, we transform raw price into normalised price with the value range of [-1,1] to improve forecasting efficiency and accuracy.

**Fig. 1:**
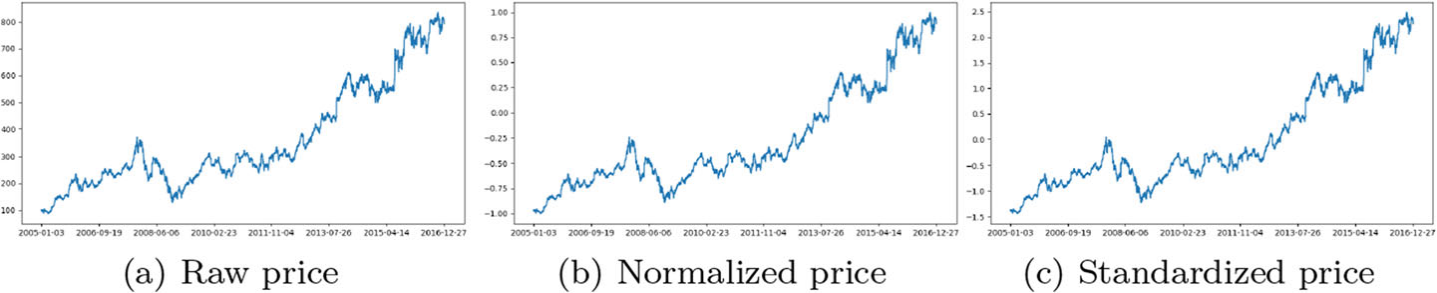
Raw price, normalized price, and z-score standardised price of Google

Furthermore, we standardise the price to calculate distance matrices between stock price time series, which can scale down the raw prices to a comparable level for clustering purpose. The z-score standardised price is defined by (2):

2 $$ \tilde{p_{t}}=\frac{p_{t}-\mu}{\sigma},  $$

where *μ* and *σ* are the mean and standard deviation of the price time series. In order to demonstrate the above price transformations, we take the Google stock price time series from 2005 to 2016 as an example to show its raw price in Figure 1a and the transformations by (1) and (2) in Figure 1(b) and (c), respectively.

In addition, we utilise daily log returns for individual stocks to identify their similarities. The reason is that for comparability of time series analysis, log return is able to capture the compounding effect of return growth, which is also named as geometric Brownian motion in quantitative finance. Thus, in this study, we use daily log returns to discover the return distribution of individual stocks, and then design an appropriate weight function to reflect such relative importance of price observations in similarity measure WDTW. Based on raw price time series *P*, daily log return is calculated as:.

3 $$ R_{t}=\ln p_{t}-\ln p_{t-1},  $$

#### Problem statement

Given stock daily price time series with a set of observations over *t* time intervals, we aim to predict stock price on day *t* + 1 with clustering-enhanced prediction framework by minimising the differences between predicted price and actual price.

### Prediction framework of stock price

**Fig. 2:**
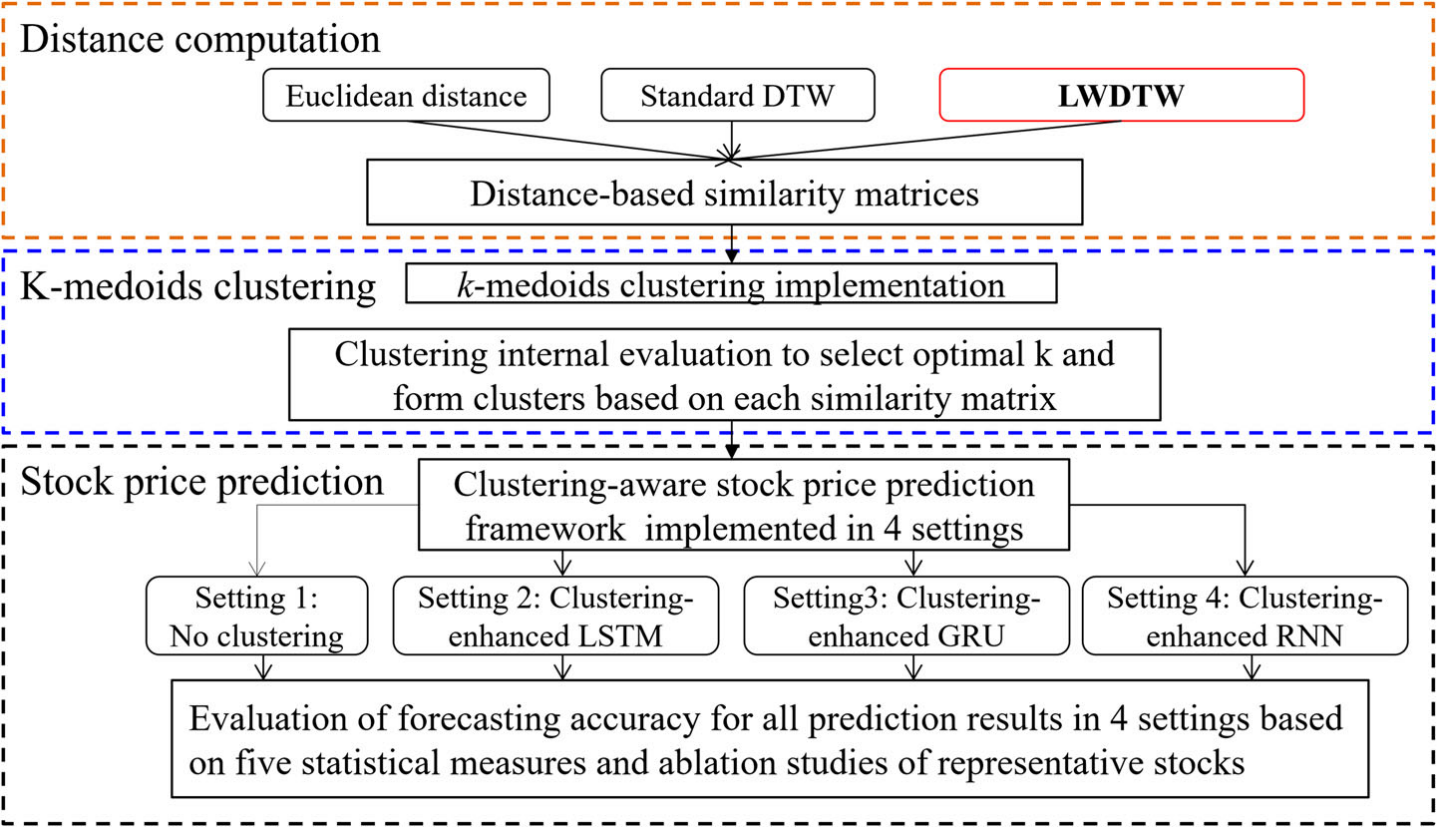
The framework of this study

The key idea of the framework is to incorporate clustering into stock price prediction framework with deep learning models in different settings. Here, three settings are customized with three different deep learning models, and one setting is conducted without clustering information. That is, with clustering-enhanced strategy, clustering is implemented with three different similarity measures in each deep learning model prediction setting. Thus, there are 4 ∗ 3 sub-settings in total under this framework as shown in Figure 2.

As shown in Figure 2, the framework consists of three main steps^1^:

- Step 1: Similarity computation
  In order to implement an efficient clustering task for stock price time series, we propose a new Weighted Dynamic Time Warping (WDTW), called Logisitc WDTW (LWDTW) to identify similar stocks with similar price patterns. For comparison purpose, two well-known distance-based similarity measures, including Euclidean distance and standard DTW, are used as benchmark distance functions.
- Step 2: *k*-medoids clustering
  Based on the above three similarity measures, we implement *k*-medoids clustering method to group similar stocks into one cluster. Two commonly used clustering internal evaluation measures of Dunn Index (D) and Davies-Bouldin Index (DB) are calculated to help us select the number of optimal k and decide the formation of clusters.
- Step 3: Stock price prediction
  We use three deep learning forecasting models, Long short-term memory (LSTM) model, Recurrent Neural Network (RNN) and Gated Recurrent Unit (GRU), to predict stock prices. The above clustering process is implemented before the employment of each forecasting model. Together with no clustering settings, there are four comparable settings, named as no clustering, clustering-enhanced LSTM, clustering-enhanced GRU, and clustering-enhanced RNN settings. Furthermore, due to three similarity measures used for *k*-medoids clustering, there are three sub-settings within each setting. Therefore, there are 12 sub-settings in total for comparable stock price predictions.

### Similarity computation

In order to implement clustering algorithms, the first step is to calculate distance matrices between stock price time series. In particular, in this study, we use z-score standardised price time series data $\tilde {P}$ as shown by Equation (2): to measure distances between two individual stocks *m* and *n*. There are two benchmark distance functions. The simplest distance function is the Euclidean distance, shown by (4):

4 $$ d(\tilde{P}^{m},\tilde{P}^{n})=\sqrt{(\tilde{p}_{1}^{m}-\tilde{p}_{1}^{n})^{2}+(\tilde{p}_{2}^{m}-\tilde{p}_{2}^{n})^{2}+...+(\tilde{p}_{t}^{m}-\tilde{p}_{t}^{n})^{2}}  $$

where *t* is the length of price time series, and $\tilde {p}_{i}^{m}$ and $\tilde {p}_{i}^{n}$ are the *i*-th observations of time series $\tilde {P}^{m}$ and $\tilde {P}^{n}$, respectively.

Another benchmark model is Dynamic Time Warping (DTW), to obtain optimal matches by using smallest distances between time series. Let $c(\tilde {p}_{i}^{m}, \tilde {p}_{j}^{n})$ be the cost function to measure the dissimilarity between $\tilde {P}^{m}$ and $\tilde {P}^{n}$ and *w* be the weight of an allowed path, thus

5 $$ w(p)={\sum}_{i,j\in P} \ c({\tilde{P}_{i}^{m}, \tilde{P}_{j}^{n}})\  $$

where P is the set of possible warping paths, so it is represented as *p* ∈ *P* and there are numerous possible warping paths p to match two time series.

#### **Definition 2**

Given a cost function, the DTW distance between $\tilde {P}^{m}$ and $\tilde {P}^{n}$ time series is defined as:

6 $$ {D(\tilde{P}^{m},\tilde{P}^{n})}=\min_{p\in P}w(p)  $$

To simplify the notation, we use *D*_*mn*_ as the DTW distance between two price time series, that is, $D_{mn}={D(\tilde {P}^{m},\tilde {P}^{n})}$ Similarly, the cost function $c({\tilde {P}_{i}^{m}, \tilde {P}_{j}^{n}})$ is denoted as *C*_*mn*_. One path p will be selected as the optimal path to calculate similarity between time series when minimised *D*_*mn*_ is achieved. Hence, the shorter the distances between price time series, the more similar stocks are. The solution to (6) can be realised by using optimization techniques with dynamic programming. The the optimal path can be found by the recursive (7):

7 $$ D_{mn}=C_{mn}+\min{\bigl\{D_{m-1, n-1}, D_{m-1, n}, D_{m,n-1}}\bigr\}  $$

Obviously, the Euclidean distance is derived from spatial proximity and is easy to use even for some large data sets [45]. Nevertheless, its assumption that time series have equal length and time axis should be not warped for best alignments does not always hold for real-world applications. Hence, by allowing time axis warping, standard DTW method is proposed to obtain optimal matches by using smallest distances between time series. However, DTW method assumes that all alignments are equally important and are given equal treats when calculating distance matrix between time series.

To address this issue by considering relative importance of each alignment, weighted DTW (WDTW) model has been proposed and successfully applied to some real world applications. The reason is WDTW introduces parameters in new weighting functions to control the penalization or favour of different contributions between alignments. In this study, we extend this key idea of WDTW model to stock price time series.

In order to identify similar patterns of individual stocks, we have visualised the distributions of daily log returns for all 322 sample stocks. Consistent with our common sense and previous empirical findings of stock return distributions [12], we have observed that individual stock returns always have more frequently extreme observations of high and low values than they should be in normal distribution case. This leads to the idea that logistic probability density distribution could be used as another possible representative distribution for our sample stocks, which has higher peaks, fatter tails and a bit asymmetric shape. Therefore, the probability density function of logistic distribution is selected as the appropriate weight function to modify the distance matrices between price time series by under-weighting the contributions of extreme observations, but emphasizing the importance of ordinary observations. The new proposed WDTW is called Logistic WDTW (LWDTW) and is given by (8):

8 $$ l(R_{i}, \mu, s)=\frac{e^{\frac{-(R_{i}-\mu)}{s}}}{s(1+e^{\frac{-(R_{i}-\mu)}{s}})^{2}}  $$

where *l*(*R*_*i*_,*μ*,*s*) is the parameter of the cost function, *R*_*i*_ is the log return calculated by (3), *μ* is location parameter, and *s* is a scale parameter for the logistic distribution of stock prices. In this stock price time series setting, the weight of a path *p* cost function shown by (5) can be redefined as:

9 $$ w_{l}(p)={\sum}_{i,j\in P} \ l(R_{i}, \mu, s) c({\tilde{P}_{i}^{m}, \tilde{P}_{j}^{n}})  $$

where *w*_*l*_(*p*) is the cost parameter, calculated by substituting weight parameter presented by (8) in (5). The WDTW distance function with logistic distribution is refined as:

10 $$ {D(\tilde{P}^{m},\tilde{P}^{n})}_{l}=\min_{p\in P}w_{l}(p)\  $$

where P is the set of allowed paths. In order to solve the LWDTW optimisation problem in (10), the following dynamic programming techniques given by the recursive (11),

11 $$ D_{mn}=C_{mn}+\min \left\{ \begin{array}{lr} l_{m-1,n-1}D_{m-1,n-1}, &\\ l_{m-1,n}D_{m-1,n}, &\\ l_{m,n-1}D_{m,n-1}. & \end{array} \right.  $$

**Fig. 3:**
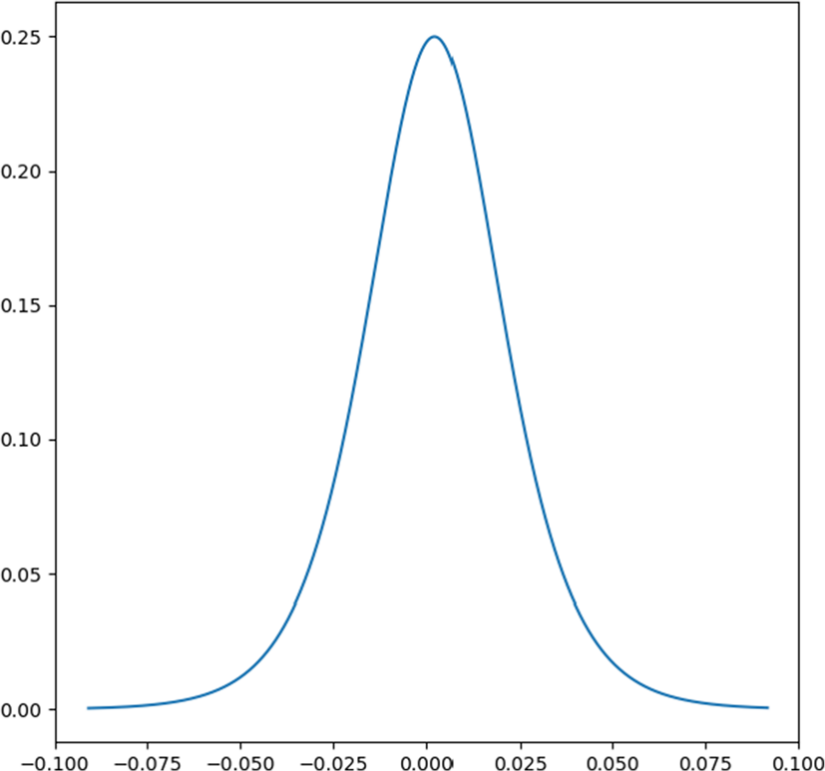
Logistic weights distribution given for individual stocks. x-axis and y-axis represent stock returns and corresponding Logistic weights, respectively

The new proposed Logistic weight matrix for each stock price has been calculated and shown in Figure 3 which also gives *μ*= 0.0004 and *s*= 0.0001.

### *K*-medoids clustering

The well-known and widely used clustering methods are the partitioning methods. The simplest partitioning methods are *k*-means and *k*-medoids clustering when the mean or median are used as centroid of a cluster respectively. In this study, we choose *k*-medoids [24] clustering method to assign similar stocks because of its simple implementation and promising results with price time series data. Other clustering methods are not appropriate. For example, the hierarchical clustering is useful to group observations at different levels and density-based clustering is good at grouping data for non-spherical or arbitrary shapes. To assess clustering results, two commonly used internal clustering evaluation indicators, Dunn Index (DI) [10] and Davies-Bouldin Index (DBI) [7] calculated by (12) and (13), are used to measure compactness and separateness for each cluster. Then the optimal number of clusters *k* is selected based on each evaluation measure. This clustering process is applied to our clustering-enhanced prediction framework.

For each cluster *C*, the Dunn Index is calculated as follows:

12 $$ DI={\frac {\min_{1\leq i<j\leq n}d(c_{i},c_{j})}{\max_{1\leq k\leq n}d^{\prime }(c)}}.  $$

Similarly, for each cluster, DB Index is calculated by the following formula:

13 $$ DBI=\frac{1}{n}{\sum}_{i=1}^{n}\max_{j\neq i}(\frac{\bar{S_{i}}+\bar{S_{j}}}{d(c_{i}, c_{j})}),  $$

where *d*(*c*_*i*_,*c*_*j*_) represents the inter-cluster distance between cluster *i* and cluster *j*, *n* is the number of clusters, *d*^′^(*c*) measures the intra-cluster distance of cluster *c*, $\bar {S_{i}}$ is the average distance of all observations in cluster *i* from its cluster mean and $\bar {S_{j}}$ is the average distance of all observations in cluster *j* from its cluster median.

### Deep learning prediction

When considering time series data, it is very likely that outputs at later time steps are somehow dependent on inputs at prior time steps. The missing relationship inherent to time series data between earlier inputs and later predictions should be captured by some neural networks that can pass old information on to future prediction at many time steps. RNN, LSTM and GRUs are designed to address the above issue, because they have internal memory to link past memory to current or future predictions. These fantastic advantages of the three models enable them to do excellent job in time series predictions. As explained in Section 3.1, normalised prices shown by (1) are used as inputs of forecasting process with different deep learning models.

#### RNN model

In the application of daily stock price prediction, the updated value of RNNs internal cell state is redefined by (14) and the expected value of output is also redefined by (15).

14 $$ h_{t}=tanh{ (W_{hh}^{T} \cdot h_{t-1} + W_{{\hat{p}_{t}}h}^{T} \cdot \hat{p}_{t})},  $$

15 $$ {E(\hat{p}_{ot})=W_{h\hat{p}_{ot}}^{T}} \cdot h_{t},  $$

where *h*_*t*_ denotes the hidden state at day *t*, *h*_*t*− 1_ denotes hidden state on day *t* − 1, $\hat {p}_{t}$ is the input at time step *t*, $E(\hat {p}_{ot})$ is the output at time step *t*, and $W_{hh}^{T}$, $W_{{\hat {p}_{t}}h}^{T}$ and $W_{h\hat {p}_{ot}}^{T}$ are the weight matrices that define the relation between the prior hidden state and current hidden state ($W_{hh}^{T}$), how the inputs $\hat {p}_{t}$ are being transformed in the hidden state ($W_{{\hat {p}_{t}}h}^{T}$), and how to transform hidden state *h*_*t*_ to output ($W_{h\hat {p}_{ot}}^{T}$), respectively.

#### LSTM model

RNN’s internal memory is a short-term memory and may lose important past information from earlier time steps when a sequence is long. This is called the vanishing gradient problem. To solve this problem, derived from standard RNNs, LSTM model has been proposed with long-term and short-term memory by using gates to control information flow and decide what information should be memorised and what information should be got rid of. There are three gates involved in LSTM model, including forget gate, input gate and output gate. LSTM is defined based on standard neural network operations, such as *sigmoid* and *tanh* nonlinear activation functions. Each gate consists of a *sigmoid* function and a pointwise multiplication operation and is introduced as follows.

- Forget gate
  LSTM uses forget gate to decide what information of previous state is not relevant and should be forgotten. Specifically, a *sigmoid* activation function is applied with inputs $\hat {p}_{t}$ and prior cell states *h*_*t*− 1_, which is expressed between 0 and 1, interpreted as how much input could be passed through between nothing or everything.
  16 $$ f_{t}=\sigma (W_{f} \cdot [h_{t-1}, {\hat{p}}_{t}]+b_{f})  $$
- Input gate
  The next step is to decide what part of new information is the most relevant and should be stored in cell state. There are two layers involved in this update process. One is *sigmoid* layer, also called the input gate layer, which decides how much of new information will be updated to cell state. The other layer is a *tanh* layer, which produces a vector of new candidate values that could be added to the state. The product of these two terms are used to update old cell state *C*_*t*− 1_ into new cell state *C*_*t*_.
  17 $$ i_{t}=\sigma (W_{i} \cdot [h_{t-1}, {\hat{p}}_{t}]+b_{i})  $$
  18 $$ \tilde{C}_{t}=tanh (W_{C} \cdot [h_{t-1}, {\hat{p}}_{t}]+b_{C})  $$
  19 $$ C_{t}= f_{t} * C_{t-1} + i_{t} * \tilde{C}_{t}  $$
- Output gate
  A *sigmoid* function layer, called output gate, is applied to control what part of the cell state should be outputted. The cell state *C*_*t*_ is put through activation function *tanh* to generate output and then the output is multiplied by the value of *sigmoid* gate *o*_*t*_. Finally, a modified output *R*_*t*_ will be generated.
  20 $$ o_{t}=\sigma (W_{o} \cdot [h_{t-1}, {\hat{p}}_{t}]+b_{o})  $$
  21 $$ h_{t}= o_{t} * tanh(C_{t})  $$

Therefore, the core intuition behind LSTM is that by using gates, information flow can be selectively added or removed through many time steps, where both relevant short-term and long-term information can be memorised. Again, this most attractive advantage about LSTM could make it become the most suitable prediction deep learning models for stock price predictions.

#### GRU model

Similar to LSTM, Gated recurrent units (GRUs) is also a gating mechanism of RNNs and has a simpler architecture to regulate flows of information. Instead of three gates in LSTM, there are two gates, reset gate and update gate, used in GRUs. Like the actions of forget gate and input gate of LSTM, the update gate of GRUs is responsible for long term memory and is able to decide what part of previous information should be forgotten and what part of new information should be added. Like the forget gate of LSTM, the reset gate is responsible for short term memory of networks and able to decide what information is irrelevant and should not be carried forward. Hence, another difference between LSTM and GRUs is that there are two states in LSTM, cell state and hidden state, but only one state -hidden state in GRU model.

## Data preparation

In this section, we describe the details about data preparation.

### Data description

**Fig. 4:**
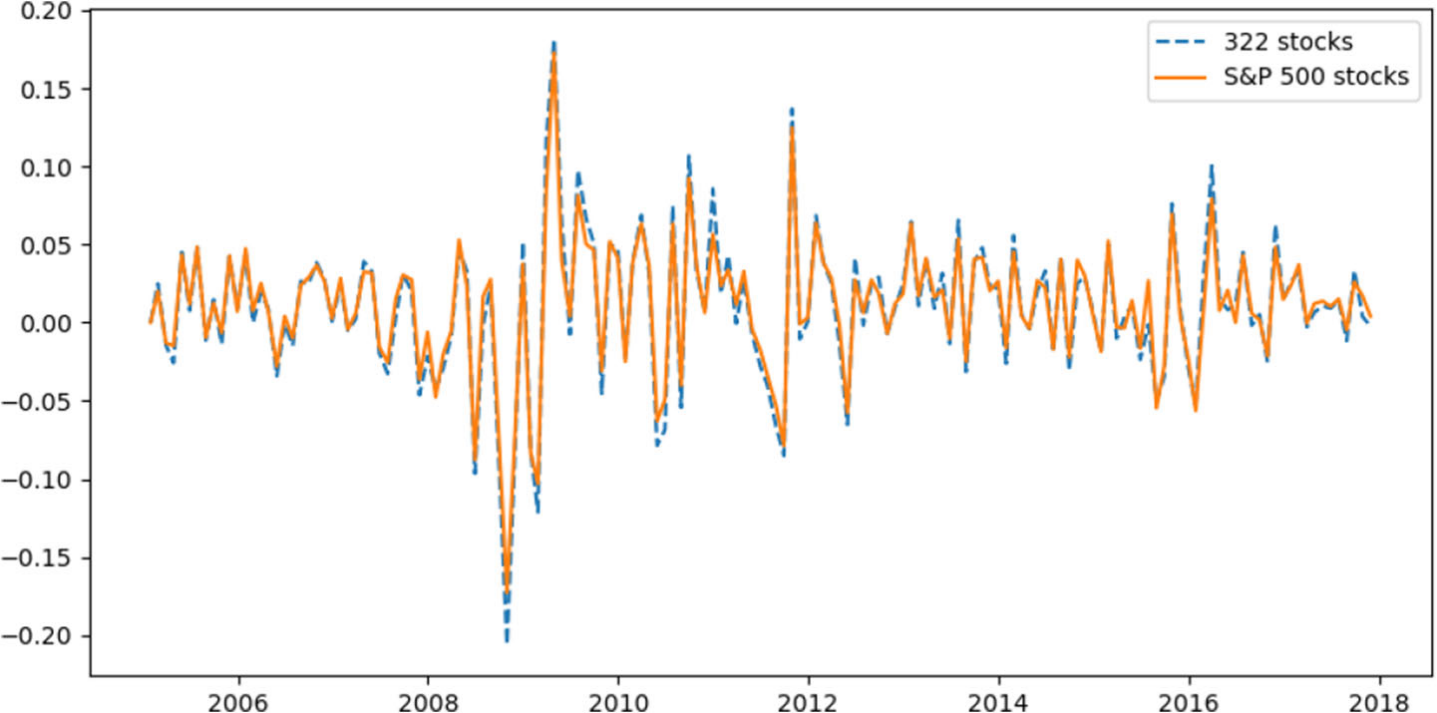
Value weighted monthly returns for our sample stocks and S&P 500 Index from 01/2005 to 11/2017, respectively. Value-weighted returns are calculated by giving a weight to a stock in the portfolio based on its market value. The stocks with higher market values have higher weights and stocks with smaller market values have lower weightings in the calculation of value-weighted returns. Market value of each stock is equal to the price of the stock multiplied by the number of shares outstanding

Due to the free availability of high-quality financial data, we choose to download historical stock prices from the public data platform Kaggle.com, which is one of the most well-known websites amongst data scientists and machine learning engineers community.^2^ The original dataset has included full historical daily prices and volume information for US stocks listed on NYSE, NASDAQ, and NYSE MKT (AMEX) from 1970-2017, such as open, high, low, close prices and trading volume. In order to examine stock price patterns in recent years, we select our sample period from 01/2005 to 11/2017. Furthermore, we keep stocks that have successfully survived over this period. The reason is that we aim to examine our clustering-enhanced forecasting frame for stock price prediction without the influence of missing and inconsistent data. Thus, there are 322 stocks and 1039738 daily observations in total, which have covered all 11 S&P sectors (Information Technology, Health Care, Financial Services, Consumer Discretionary, Communication Services, Industrials, Consumer Staples, Energy, Utilities, Real Estate, and Materials) and nearly 70*%* stocks of them are the current components of the S&P 500 Index. The sufficient data volume and broad coverage of industries used in this study make it much superior relative to most previous papers related to stock price predictions with deep learning models, whose samples are always very small with a few market indexes or several stocks [19, 27]. We show value-weighted monthly returns^3^ for our sample stocks and S&P 500 Index from 2005 to 2017 in Figure 4. The overall performance of our sample stocks is very similar to S&P 500 Index, which indicates that our sample is also a good representative for US stock market. In addition, as the daily prices in original datasets have been adjusted for dividends and splits, this enables the prices shown in our sample dataset to reflect all relevant available information, and indicates this sample is perfect for price calculation and forecasting. When doing *k*-medoids clustering, we use standardised price, shown in (2) [24].

### Dataset partitioning and pre-processing for deep learning forecasting

**Fig. 5:**
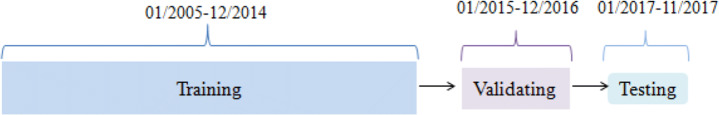
The partition of whole sample dataset for training, validating and testing

Before the implementation of deep learning forecasting models, our sample data are divided into three parts according to the ratio of 10:2:1 for training set, validation set and testing set. Specifically, as shown in Figure 5, the training, validation and test periods are set to be from 01/2005 to 12/2014, 01/2015 to 12/2016, and 01/2017 to 11/2017, respectively. The reason why we keep ten years as training period is to make our best effort to train historical stock price data and to aim at exploring more valuable information, discovering more useful price patterns, and providing more accurate forecasting results. Validation dataset is also used to further tune trained parameters with best configuration for the prediction models. For training process of LSTM or RNN or GRU networks, we use some advanced methods for better and more accurate predictions. First, Adam as an optimization algorithm to handle stochastic gradient descent problems, which has combined the best properties of RMSProp and AdaGrad. Second, the dropout regularization is used to avoid the problem with overfitting. Initially, a random fraction of input units is ignored at each update of training iterations. When it is beyond the value of 0.2, the accuracy in performance gradually decreases and loss gradually increases. So we set the dropout value of 0.2 , the batch size equal to one in stochastic mode, and the epoch equal to 15. All of them further help us find the most appropriate model for prediction with less under-fitting and over-fitting problem.

## Experiments

In this section, we demonstrate the clustering effect of our proposed similarity method, the stock price prediction of three clustering-enhanced deep learning models, and ablation studies of several selected stocks.

### Experimental setup

#### Datasets

We perform extensive experiments on stock price prediction with three deep learning models by using daily price time series for 322 US stocks over the time period of 01/2015 to 11/2017. Normalised price time series is generated by Equation (1), as described in Section 3.1.

#### Comparable methods

We implement and compare four solutions and report their results.

- Deep learning prediction without clustering: The three deep learning models, LSTM, RNN and GRU, are used to predict stock prices without the implementation of clustering before forecasting.
- Clustering-enhanced LSTM: *k*-medoids clustering, based on Logistic WDTW, Euclidean distance and Standard DTW method, is applied before forecasting. Then, we train the LSTM model within each cluster to obtain the tuned parameters, which are then used to predict individual stock prices.
- Clustering-enhanced GRU: The same process of clustering and forecasting as in the above clustering-enhancedLSTM setting is applied, but with the GRU deep learning model.
- Clustering-enhanced RNN: The same process of clustering and forecasting as in Clustering-enhanced LSTM and GRU settings is applied, but with the RNN deep learning model.

### Evaluation metrics

In order to provide reliable evaluation about forecasting accuracy, we assess the performance of prediction models by using five commonly used statistical evaluation indicators, as there is no one-size-fits-all indicator. The five measures are *Mean Absolute Percentage Error(MAPE)*, *Mean Absolute Error (MAE)*, *Mean Square Error (MSE)*, *Root Mean Square Error(RMSE)*, and *R-squared* (*R*^2^). Their definitions are given by (22)–(26).

22 $$ MAPE=\frac{1}{n}{\sum}_{t=1}^{n}\left|\frac{{p_{t+1}}-{E(p_{t+1})}}{p_{t+1}}\right|  $$

23 $$ MAE=\frac{1}{n}{\sum}_{t=1}^{n}\left|{{p}_{t+1}}-{E({p}_{t+1})}\right|  $$

24 $$ MSE=\frac{1}{n}{\sum}_{t=1}^{n} ({{p}_{t+1}}-{E({p}_{t+1})})^{2}  $$

25 $$ RMSE=\sqrt{\frac{1}{n}{\sum}_{t=1}^{n}({{p}_{t+1}}-{E({p}_{t+1})})^{2}}  $$

26 $$ R^{2}=1-\frac{{\sum}_{t=1}^{n}({{p}_{t+1}}-{E({p}_{t+1})})^{2}}{{\sum}_{t=1}^{n}({{p}_{t+1}}-{ \bar{{p}}_{t+1}})^{2}}  $$

where *E*(*p*_*t*+ 1_) is the predicted price on day *t* + 1, *p*_*t*+ 1_ is the actual price on day *t* + 1, $\bar {{p}}_{t+1}$ is the average value of all actual prices over day 1 to day *t* + 1, and *n* is the number of observations in corresponding datasets for individual stocks.

### Evaluation of clustering

Here, two common internal evaluation metrics, Dunn Index (DI) and Davies-Bouldin Index (DBI), are used to assess the quality of clustering based on their compactness and separateness. Due to the sample size, the number of clusters *k* trials from 2 to 20. The Dunn value is a ratio of the minimum inter-cluster distance divided by the maximum intra-cluster distance, hence, a higher Dunn value indicates a better quality of clustering.

**Fig. 6:**
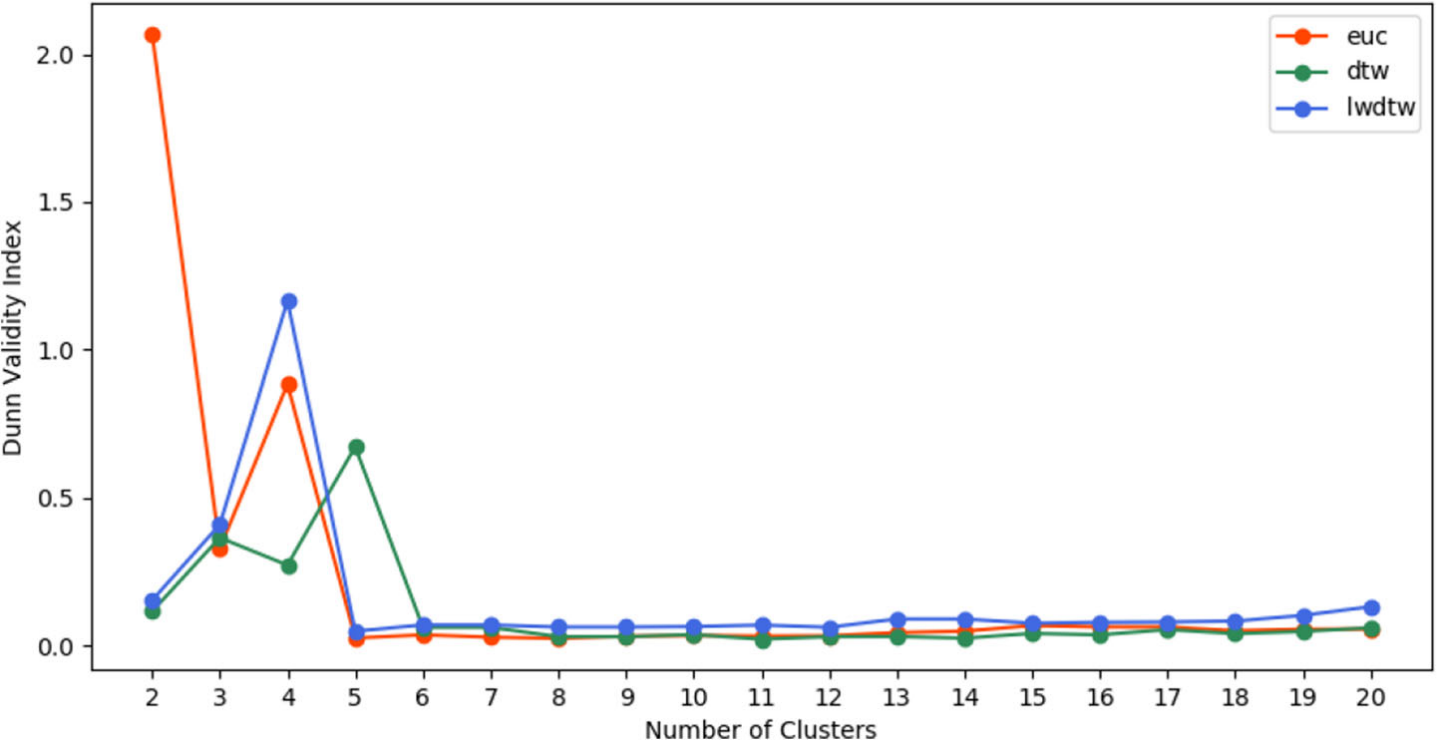
Dunn Index values for clusters when *k* is varying from 2 to 20 based on Euclidean distance (orange line), standard DTW (green line) and new proposed Logistic WDTW method (blue line), respectively

Figure 6 shows that Dunn values based on Euclidean distance, DTW and new LWDTW are obtained when different *k* values are varying from 2 to 20. It is seen that from *k* = 6, Dunn values based on all three similarities decline to 0.1 and continue to be stale at 0.1. Dunn value based on Euclidean distance has its second peak value of 0.85 at *k* = 4, and DTW Dunn value has reached its peak of 0.65 at *k* = 5. More importantly, the highest Dunn value of 1.2 has been obtained at *k* = 4 when using LWDTW measure. This suggests that LWDTW is the most effective similarity measure among the three similarity measures suggesting that the optimal cluster number is 4.

**Fig. 7:**
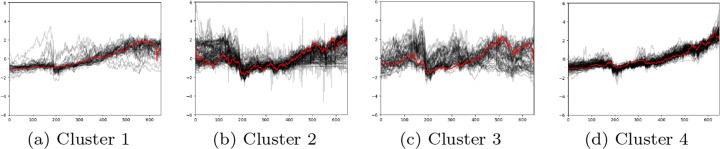
Visualisation of 4 stock clusters based on LWDTW when k is 4. X-axis and y-axis represent the number of weeks during the whole time period and z-score standardised prices of individual stocks within each cluster, respectively

Figure 7 shows 4 stock clusters based on LWDTW similarity matrix. The red line in each cluster represents the cluster barycentre from 2005 to 2017. More the stock price lines vary around cluster barycentre, more effective the similarity measure is. Hence, in Figure 7, we can see that based on LWDTW, there are 3 out of 4 clusters where red lines are good representations for the corresponding clusters.

**Fig. 8:**
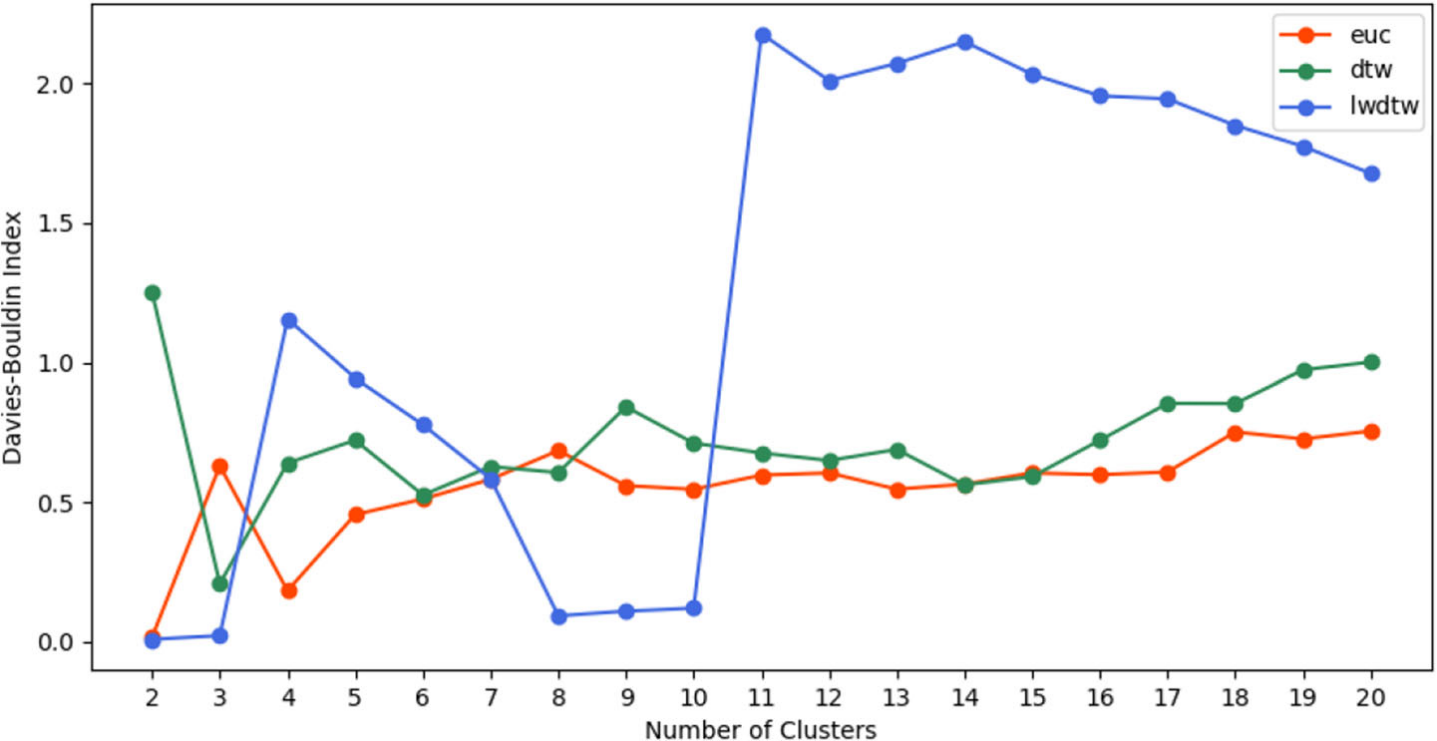
Davies-Bouldin Index values for clusters based on Euclidean distance(orange line), standard DTW (green line) and new LWDTW (blue line), respectively, when *k* is varying from 2 to 20

The DBI is another measure to evaluate the clustering effectiveness, which also captures how the clusters are well separated from each other and the compactness of stocks in one cluster. The same process of Dunn values is also implemented to generate DB values. The lower DB values indicate better quality of clustering, as indicated by (13). As shown in Figure 8, when *k* is 3, LWDTW based DB value has reached its lowest point of 0.03, DTW based DBI value is 0.2 and Euclidean distance based DB value is 0.6. When *k* increases from 5 to 20, all DTW and Euclidean distance based DB values are above 0.5 and LWDTW based DB values reach the second lowest points around 0.1 at *k*= 8, 9 and 10, which is much lower than 0.5. This strongly indicates that the new proposed LWDTW similarity measure provides more reliable performances to produce better clustering results than the other two benchmark similarity measures.

**Fig. 9:**
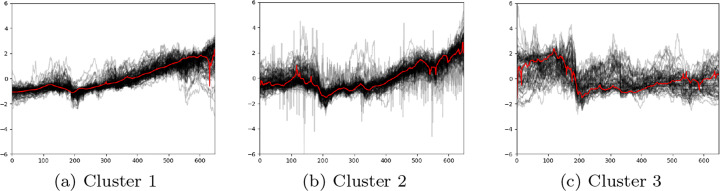
Visualisation of 3 clusters of stocks based on LWDTW when *k* is 3. X-axis and y-axis represent the number of weeks during the whole time period and z-score standardised prices of individual stocks within each cluster, respectively

In order to present clustering results, we have visualised the three LWDTW clusters in Figure 9. It is obvious that price patterns in these three clusters are very different from each other. Cluster 1 stocks start with a poor performance in 2005, but keep a upward price trend over the whole period only with a slight decline in 2008 due to global financial crisis (GFC). Compared to Cluster 1 stocks, Cluster 2 stocks have a better performance in 2005, sharper and higher decline in 2007 and 2008 due to GFC, and higher volatility recovery until 2017. In contrast to Cluster 1 and Cluster 2 stocks, Clusters 3 stocks have the opposite price trend over the whole time period with the best performances before GFC, but with the deepest price decline over GFC time period and no significant recovery occurred after that. These observations strongly indicate that the proposed LWDTW similarity measure does an excellent job in identifying similar stocks with similar price patterns. That is, based on LWDTW, it is successful to assign similar stocks within one cluster, which are also dissimilar to stocks in other clusters. The number of optimal clusters *k* is determined as 3, consistent with the findings based on Dunn Index in Figure 6, where 3 out of 4 clusters are good representatives.

### Evaluation of stock price prediction

Here, we present extensive forecasting evaluation results of stock price predictions by applying time-series deep learning forecasting models, including LSTM, RNN and GRU models. The rule of using MAE, MAPE, MSE and RMSE is that the lower values mean less prediction errors and higher prediction accuracy, whereas high values of *R*^2^ indicate less errors and high accuracy.

#### Prediction results without clustering

In order to give a detailed description of all 322 sample stocks forecasting accuracy without clustering, we provide the averages of the five evaluation measures for training, validating and testing datasets with LSTM model, GRU model and RNN model in Table 1. For example, when looking into averages of evaluation measures for LSTM model predictions, we find that training datasets have the lowest averages of MAE, MAPE, MSE, RMSE and highest averages of *R*^2^, validating datasets take the second place, and the testing datasets have the highest averages of MSE, MAPE, MAE and lowest *R*^2^. This evidence shows that our trained predicted prices are nearly the same as the actual prices and thus these trained parameters are very reliable to be used in prediction process. Furthermore, when only looking into averages of testing datasets in these three tables, we can compare the averages of each evaluation measure for three models, and find that the LSTM model has the best forecasting performance with the lowest averages of MAE 0.0774, MAPE 0.1706, MSE 0.0133, RMSE 0.0984 and highest averages of *R*^2^ 0.9047, whereas GRU model has the worst performance with corresponding averages of 0.4843, 0.6989, 0.5746, 0.2026 and -2.7541. This indicates that for the No clustering forecasting strategy, LSTM model has the best predictive ability among the three models to forecast stock prices.

**Table 1:** Forecasting accuracy for LSTM, GRU and RNN models

		MAE	MAPE	MSE	RMSE	*R*^2^
LSTM	Training	0.0260	0.2553	0.0013	0.0350	0.9929
	Validating	0.0569	0.5103	0.0065	0.0780	0.9678
	Testing	0.0774	0.1706	0.0133	0.0984	0.9047
GRU	Training	0.1296	0.8299	0.0398	0.5427	0.7960
	Validating	0.1534	1.1294	0.0527	0.1747	0.7519
	Testing	0.4843	0.6989	0.5746	0.2046	-2.7541
RNN	Training	0.0239	0.2729	0.0012	0.0329	0.9937
	Validating	0.0552	0.5840	0.0036	0.0752	0.9703
	Testing	0.1431	0.2438	0.0725	0.1826	0.8087

#### Prediction results with clustering

In contrast to the above No clustering forecasting strategy, clustering-enhanced forecasting strategy is implemented within each cluster to produce trained parameters and then the predicted prices of individual stocks are generated by deep learning models with trained parameters of their corresponding clusters. In another word, the difference between these two strategies is how trained parameters are produced, by their own normalised price time series or by their corresponding cluster-related time series. The key logic behind these two strategies is that the more reliable trained parameters are used for prediction, the more accurate forecasting results will be produced.

As shown in Table 2 to Table 6, we select 70 out of 322 stocks to forecast stock prices, which are all S&P 500 components and cover all 11 sectors. In order to give more description of forecasting accuracy for these 70 stocks, we present the average of each evaluation measure for top 10, top 20, top 30, top 40, top 60 and all 70 stocks in columns. As discussed in Section 5.3, the optimal number of clusters *k* is 3, so we only specify *k* as 3 in the following evaluation.

**Table 2:** Averages of MAPE to measure three deep learning models’ forecasting accuracy for 70 US stocks under clustering-enhanced settings based on three similarity measures, that is, Euclidean distance (EUC), standard DTW and LWDTW respectively

	MAPE Averages	ALL 70	Top 10	Top 20	Top 30	Top 40	Top 50	Top 60
LSTM	Without clustering	0.1385	0.0327	0.0395	0.0462	0.0553	0.0693	0.0899
	**LWDTW**	**0.1278**	**0.0280**	**0.0322**	**0.0362**	**0.0421**	**0.0541**	**0.0765**
	EUC	0.1296	0.0335	0.0381	0.0428	0.0480	0.0581	0.0813
	DTW	0.1281	0.0327	0.0373	0.0414	0.0469	0.0579	0.0804
GRU	Without clustering	0.5001	0.1389	0.1775	0.2261	0.2799	0.3350	0.4090
	LWDW	0.2463	*0.1140*	0.1311	0.1465	0.1576	0.1672	0.1885
	*EUC*	*0.2065*	0.1150	*0.1239*	*0.1343*	*0.1422*	*0.1500*	*0.1658*
	DTW	0.3925	0.2357	0.2574	0.2685	0.2780	0.2915	0.3262
RNN	Without clustering	0.2132	0.0611	0.0782	0.0918	0.1091	0.1289	0.1548
	*LWDTW*	*0.1830*	*0.0453*	*0.0523*	*0.0588*	*0.0699*	*0.0893*	*0.1200*
	EUC	0.3171	0.1850	0.2103	0.2251	0.2367	0.2515	0.2714
	DTW	0.3430	0.1065	0.1523	0.1854	0.2057	0.2245	0.2537

The lowest MAPE averages among all three models are presented in bold, while the lowest MAPE averages for the other two models are given in italics

In Table 2, MAPE averages are shown for LSTM, GRU, RNN models without clustering, clustering based on Euclidean distance (EUC), standard DTW and the new LWDTW forecasting models. For example, within each forecasting model, each row presents MAPE averages for all 70 stocks, and for stocks from Top 10 to Top 60. The 10 stocks with the highest forecasting accuracy (lowest MAPE averages) are grouped as Top 10 stocks and the same sorting process is applied to Top 20 to Top 60. Therefore, we can see in the first row without clustering strategy for LSTM model, the MAPE averages increase from 0.0327 (Top 10) to 0.0899 (Top 60) until 0.1385 (ALL 70) in each row. Moreover, when comparing all averages by column, we observe that clusters based on LWDTW provide the lowest MAPE averages in all corresponding columns. For LSTM model, we can see in each column, MAPE averages based on LWDTW clustering forecasting (in second row) are the lowest (0.0280 for Top 10 and 0.1278 for all 70) compared to corresponding averages in other rows. This evidence has strongly supported our above logic by showing that LWDTW is more effective to assign similar stocks into clusters and then more accurate trained parameters are produced, which finally leads to less forecasting errors with low MAPE averages. That is, with the same forecasting model, LWDTW clustering based predictions has the best performance (denoted in bold in Table 2). When comparing the averages with the same clustering formation but with different forecasting models, we find that in each column, LSTM model provides the lowest MAPE averages in corresponding rows.

Similar findings are also observed with the averages of MAE, MSE and RMSE presented in Tables 3, 4 and 5, respectively. The averages of *R*^2^ are shown in Table 6 where higher averages of *R*^2^ mean better forecasting accuracy. The results in Table 6 show that LWDTW clustering forecasting process has achieved the best performances within each forecasting model (in second row of *R*^2^ under each model), compared to *R*^2^ averages in other corresponding rows. For instance, LWDTW LSTM model has the best forecasting performances with the highest *R*^2^ averages of 0.9890 for Top 10 and 0.9517 for all 70 stocks. Even though GRU model has the worst performance compared to the other two models, when we look into *R*^2^ averages for GRU model, LWDTW clustering has achieved the highest *R*^2^ of 0.9325 for Top 10 and 0.7128 for All 70.

**Table 3:** Averages of MAE to measure three deep learning models’ forecasting accuracy for 70 US stocks under clustering-enhanced settings based on three similarity measures, i.e., Euclidean distance (EUC), standard DTW and LWDTW, respectively

	MAE Averages	ALL 70	Top 10	Top 20	Top 30	Top 40	Top 50	Top 60
LSTM	Without clustering	0.0694	0.0378	0.0416	0.0442	0.0476	0.0514	0.0577
	**LWDTW**	**0.0536**	**0.0378**	**0.0409**	**0.0433**	**0.0454**	**0.0475**	**0.0497**
	EUC	0.0609	0.0388	0.0421	0.0447	0.0471	0.0497	0.0545
	DTW	0.0589	0.0378	0.0421	0.0449	0.0473	0.0497	0.0531
GRU	Without clustering	0.3970	0.0636	0.0811	0.1036	0.1412	0.1984	0.2822
	LWDTW	0.1783	0.0674	0.0899	0.1063	0.1245	0.1407	0.1586
	*EUC*	*0.1661*	*0.0583*	*0.0689*	*0.0782*	*0.0947*	*0.1146*	*0.1371*
	DTW	0.3232	0.0910	0.1298	0.1616	0.1953	0.2338	0.2707
RNN	*Without clustering*	0.1305	*0.0402*	*0.0444*	*0.0477*	*0.0530*	*0.0687*	*0.0910*
	LWDTW	*0.1137*	0.0621	0.0695	0.0734	0.0763	0.0795	0.1036
	EUC	0.2759	0.0645	0.0895	0.1105	0.1410	0.1815	0.2242
	DTW	0.2406	0.0636	0.0852	0.1093	0.1368	0.1719	0.2071

The lowest MAE averages among all three models are presented in bold, while the lowest MAE for the other two models are in given italics

**Table 4:** Averages of MSE to measure three deep learning models’ forecasting accuracy for for 70 US stocks under clustering-enhanced settings based on three similarity measures, that is, Euclidean distance (EUC), standard DTW and LWDTW respectively

	MSE Averages	ALL 70	Top 10	Top 20	Top 30	Top 40	Top 50	Top 60
LSTM	Without clustering	0.0100	0.0027	0.0033	0.0037	0.0043	0.0051	0.0064
	**LWDTW**	**0.0059**	0.0028	**0.0032**	**0.0035**	**0.0039**	**0.0043**	**0.0048**
	EUC	0.0077	0.0028	0.0033	0.0036	0.0041	0.0047	0.0058
	DTW	0.0073	**0.0027**	0.0033	0.0038	0.0042	0.0047	0.0055
GRU	Without clustering	0.5251	0.0073	0.0124	0.0232	0.0458	0.0969	0.2231
	LWDTW	*0.0440*	0.0080	0.0129	0.0172	0.0227	0.0279	0.0349
	EUC	0.0481	*0.0057*	*0.0078*	*0.0101*	*0.0149*	*0.0214*	*0.0312*
	DTW	0.1656	0.0134	0.0258	0.0392	0.0552	0.0794	0.1070
RNN	Without clustering	0.0805	*0.0032*	*0.0040*	*0.0047*	*0.0072*	0.0148	0.0287
	LWDTW	*0.0124*	0.0064	0.0075	0.0081	0.0088	*0.0097*	*0.0108*
	EUC	0.1316	0.0077	0.0142	0.0208	0.0330	0.0539	0.0835
	DTW	0.0861	0.0068	0.0111	0.0188	0.0301	0.0458	0.0648

The lowest MSE averages among all three models are presented in bold, while the lowest MSE for the other two models are given in italics

**Table 5:** Averages of RMSE to measure three deep learning models’ forecasting accuracy for for 70 US stocks under clustering-enhanced settings based on three similarity measures, i.e., Euclidean distance (EUC), standard DTW and LWDTW respectively

	RMSE Averages	ALL 70	Top 10	Top 20	Top 30	Top 40	Top 50	Top 60
LSTM	Without clustering	0.0909	0.0529	0.0571	0.0608	0.0648	0.0700	0.0772
	**LWDTW**	**0.0745**	**0.0513**	**0.0554**	**0.0588**	**0.0619**	**0.0651**	**0.0683**
	EUC	0.0830	0.0524	0.0566	0.0599	0.0636	0.0674	0.0740
	DTW	0.0813	0.0517	0.0571	0.0608	0.0642	0.0679	0.0728
GRU	Without clustering	0.5311	0.0850	0.1081	0.1418	0.1893	0.2606	0.3696
	LWDTW	0.1963	0.0870	0.1097	0.1265	0.1442	0.1594	0.1768
	*EUC*	*0.1927*	*0.0748*	*0.0872*	*0.0983*	*0.1163*	*0.1365*	*0.1607*
	DTW	0.3567	0.1110	0.1529	0.1872	0.2207	0.2603	0.2993
RNN	Without clustering	0.2077	*0.0563*	*0.0620*	*0.0673*	*0.0802*	0.1061	0.1399
	LWDTW	*0.1093*	0.0796	0.0860	0.0896	0.0935	*0.0979*	*0.1027*
	EUC	0.3097	0.0869	0.1151	0.1379	0.1691	0.2088	0.2540
	DTW	0.2613	0.0817	0.1026	0.1295	0.1600	0.1936	0.2279

The lowest RMSE averages among all three models are presented in bold while the lowest RMSE for the other two models are given in italics

**Table 6:** Averages of *R*^2^ to measure three deep learning models’ forecasting accuracy for 70 US stocks under clustering-enhanced settings based on three similarity measures, i.e., Euclidean distance (EUC), standard DTW, Logistic WDTW method (LWDTW), respectively

	*R*^2^ Averages	ALL 70	Top 10	Top 20	Top 30	Top 40	Top 50	Top 60
LSTM	Without clustering	0.9374	0.9840	0.9796	0.9747	0.9686	0.9623	0.9542
	**LWDTW**	**0.9517**	**0.9890**	**0.9859**	**0.9828**	**0.9789**	**0.9739**	**0.9653**
	EUC	0.9472	0.9850	0.9802	0.9750	0.9706	0.9657	0.9592
	DTW	0.9475	0.9847	0.9812	0.9812	0.9722	0.9673	0.9591
GRU	Without clustering	-1.5685	0.9203	0.8671	0.8020	0.6743	0.3623	-0.2170
	LWDTW	0.7128	0.9325	0.9100	0.8884	*0.8680*	*0.8373*	0.7951
	EUC	*0.7532*	*0.9349*	*0.9183*	*0.8936*	0.8665	0.8349	*0.8007*
	DTW	0.1518	0.8240	0.7357	0.7357	0.5423	0.4468	0.3381
RNN	Without clustering	0.6968	0.9712	0.9606	0.9446	0.9250	0.8974	0.8318
	*LWDTW*	*0.8962*	*0.9790*	*0.9694*	*0.9618*	*0.9540*	*0.9416*	*0.9267*
	EUC	0.3155	0.9079	0.8643	0.7959	0.7141	0.6041	0.4779
	DTW	0.5449	0.9244	0.9244	0.8707	0.7586	0.7024	0.6383

The highest *R*^2^ averages among the three models are presented in bold while the highest *R*^2^ for the other two models are given in italics

Thus, based on the results from Table 2 to Table 6, clustering LWDTW LSTM model provides the best forecasting accuracy results among all forecasting models shown by the averages of the evaluation measures in corresponding rows. The new proposed similarity measure LWDTW is not only useful to efficiently assign similar stocks into clusters, but also to effectively contribute to improving the deep learning forecasting accuracy for all three deep learning models.

### Ablation studies of representative stocks

Besides the extensive evaluation results in Section 5.4, we further select six well-known stocks from six sectors, including Johnson & Johnson (healthcare), Google (Communication Services), Moody (Financial Services), Pepsi (Consumer Defensive), Microsoft (Technology), NIKE (Consumer Cyclical), as representatives to visualise forecasting performances with three deep learning models in Table 7. The US stock market in 2017 is boosted by strong economic growth, solid company earnings and governments’ tax cuts. Prices for five out of six stocks have surged and reached their records by keeping an upward trend throughout the whole year, such as Johnson and Johnson, Google, Microsoft, Moody and Pepsi. Unlike the other 5 selected stocks, prices of NIKE stock haven’t kept an upward trend but with a fluctuation. This is caused by the challenges and issued facing by NIKE, such as restructuring plan, falling sales in North America, decrease of market shares with strong market rivals, etc.

**Table 7:**
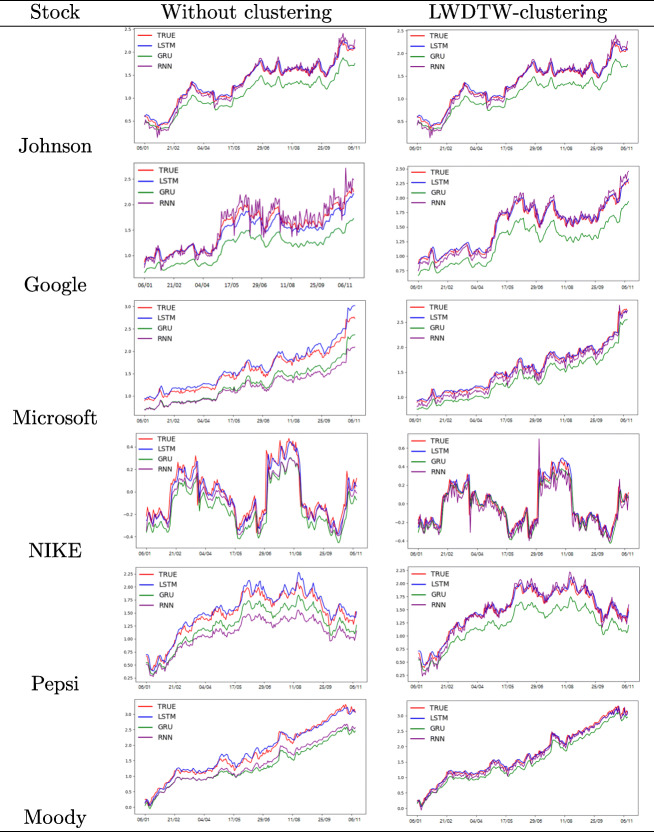
Predicted prices for 6 selected stocks are presented in blue lines (LSTM), purple lines (RNN) and green lines (GRU) with LWDTW clustering-enhanced forecasting setting as well as without clustering setting over the time period of 01/2017 to 11/2017. Actual prices are presented by red lines

In Table 7, the second column gives the forecasting result without clustering and the third column with LWDTW-clustering. In each figure, we illustrate the actual prices of each stock and their corresponding predicted prices given by LSTM, RNN and GRU models, represented with red, blue, purple and green lines respectively in both columns. Hence, there are three predicted price lines and one actual price line in each figure. The farther predicted price line from the actual price line means a worse forecasting performance for that forecasting model. As shown, the LSTM model predicted line (blue) is the closest to the actual price line (red) for each stock in both columns, which suggests again that LSTM model has the best predictive ability among the three models. In addition, to emphasize the influence of the similarity measures on the forecasting, we compare forecasting results without clustering setting and LWDTW-clustering settings with the three deep learning models. The reason is that the above evaluation results show that LWDTW-based clustering with LSTM model has the lowest forecasting errors and highest forecasting accuracy. Therefore, when looking into a stock, we can compare the same colour of predicted lines for corresponding model in both columns and find that all three different colour lines become closer to the red line in the second column than them in the first column. This evidence suggests that LWDTW-based clustering prediction has improved forecasting accuracy for all three deep learning models.

## Conclusion

In this paper, we present a clustering-enhanced prediction framework with deep learning models for stock price time series. Aiming at a high accuracy of deep learning forecasting, we incorporate stock clustering into price prediction framework and also use four comparable settings to implement the framework. Specifically, in order to conduct an effective clustering, we propose a novel weighted DTW similarity measure with logistic distribution probability density function, called LWDTW, to assign similar stocks into clusters. Compared to distance-based benchmark similarity measures, Euclidean distance and standard DTW method, LWDTW has successfully identified similar stocks within one cluster and achieved the best clustering results, as it has recognized the relative importance of price observations by giving favours or penalties to cost function of distance matrices. In addition, we use three commonly used deep learning forecasting models to conduct prediction, LSTM, RNN and GRU models. Hence, together with ”Without clustering” forecasting setting, there are four comparable settings to implement our proposed framework. Within each setting, as clustering is employed based on three different similarity matrices, there are 12 sub-settings in total in this study.

We conduct extensive experiments by using real-world stock price datasets to demonstrate the performance of three prediction models within the framework using five well-known evaluation metrics. The evaluation results show that LWDTW-based clustering prediction with LSTM model has achieved the best estimates of stock prices with the lowest averages of MAPE (0.1278), MAE (0.0536), MSE (0.0059), RMSE (0.0745) and highest average of *R*^2^ 0.9517. The reason is that when more similar stocks are identified and assigned into one cluster, the forecasting training process will learn more accurate parameters within one cluster. When those trained parameters are used to forecast stock prices, better estimates will be made. Hence, we conclude that the new proposed LWDTW similarity measure has successfully recognized similar stocks, effectively influenced clustering effect, then led to more accurate estimates of predicted prices. The proposed prediction framework can be used in real applications where it assists investors with making optimal investment decisions.

## Footnotes

1 Regarding the inputs of this framework, Z-score standardised prices (based (2)) are used for distance computation and clustering algorithms. The normalised prices (based on (1)) are used in the prediction process. In particular, log returns are used to calculate the weight functions for LWDTW method.

2 https://www.kaggle.com/borismarjanovic/price-volume-data-for-all-us-stocks-etfs

3 Value-weighted portfolio returns are the sum of product of weights and respective stock returns, where the weight is given by the proportion of market value for each stock to total market value of that portfolio. That is, the stocks with higher market values have higher weights and stocks with smaller market values have lower weightings in the calculation of value-weighted returns. Market value of each stock is equal to the price of the stock multiplied by the number of shares outstanding.
